# Psychometric Analysis of the Hip Disability and Osteoarthritis Outcome Score (HOOS)

**DOI:** 10.3390/healthcare12171789

**Published:** 2024-09-07

**Authors:** Emilie N. Miley, Madeline P. Casanova, Michael A. Pickering, Scott W. Cheatham, Lindsay W. Larkins, Adam C. Cady, Russell T. Baker

**Affiliations:** 1 Institute of Sports Sciences and Medicine, Department of Health, Nutrition and Food Sciences, Florida State University, Tallahassee, FL 32306, USA; emilie.miley1@gmail.com; 2 Tallahassee Orthopedic Clinic, Tallahassee, FL 32308, USA; 3 WWAMI Medical Education Program, University of Idaho, Moscow, ID 83844, USA; mcasanova@uidaho.edu; 4 Idaho Office of Underserved and Rural Medical Research, University of Idaho, Moscow, ID 83844, USA; 5 Department of Movement Sciences, University of Idaho, Moscow, ID 83844, USA; michaela@tonypickering.com (M.A.P.); scheatham@uidaho.edu (S.W.C.); lindsayw.usc@gmail.com (L.W.L.); 6 Kaiser Permanente, Woodland Hills, Los Angeles, CA 91367, USA; adam.c.cady@kp.org

**Keywords:** total hip arthroplasty, confirmatory factor analysis, multi-group invariance testing, latent growth-curve modeling

## Abstract

Hip Disability and Osteoarthritis Outcome Survey (HOOS) was developed as a region- and disease-specific outcome to assess hip disability. Despite the use of the HOOS in clinical practice and research, psychometric analyses of the scale in a large dataset of patients have not been performed. As such, the purposes of this study were to assess the structural validity of the HOOS in patients who underwent a total hip arthroplasty. Data were obtained from the Surgical Outcome System (SOS) global registry. Confirmatory factor analysis (CFA) was conducted to assess the scale structure of the 40-item HOOS and exploratory factor analysis (EFA) was conducted to identify a parsimonious scale structure. The parsimonious model identified was subjected to multi-group and longitudinal invariance testing and LGC modeling. The original five-factor, 40-item HOOS did not meet recommended model fit indices values (CFI = 0.822, TLI = 0.809, IFI = 0.822, RMSEA = 0.085). Alternate model generation identified an alternative model (i.e., HOOS-9). Sound model fit was identified for the HOOS-9 (CFI = 0.974, TLI = 0.961, RMSEA = 0.046). Invariance testing criteria were also met between groups (i.e., age and sex) and across time. Lastly, a nonlinear growth trajectory was identified in responses pertaining to hip disability. The original scale structure of the 40-item HOOS was not supported. The HOOS-9 met contemporary model fit recommendations, along with multi-group and longitudinal invariance testing. Our findings support the preliminary use of the HOOS-9 to assess hip function and disability in research and clinical practice.

## 1. Introduction

Hip Disability and Osteoarthritis Outcome Survey (HOOS) was developed as a region- and disease-specific outcome to assess disability pertaining to osteoarthritis (OA) [1]. The HOOS development process relied heavily on two previously developed instruments: (1) the Western Ontario McMaster Osteoarthritis Score (WOMAC), and (2) the Knee Injury and Osteoarthritis Outcome Score (KOOS). The WOMAC is a disease-specific instrument validated for OA in the lower extremities and for evaluating outcomes after a total hip arthroplasty (THA) [1,2], while the KOOS is a region-specific instrument intended to measure pain, symptoms, activities of daily living (ADLs), sport and recreation function, and knee-related quality of life (QOL) in middle aged patients with or without knee OA [3]. The HOOS contains items (n = 24) and proposed constructs (i.e., pain, stiffness, physical function) of the WOMAC [1,4]; the HOOS also contains items (n = 11) and proposed constructs (i.e., sport and recreational function, QOL) derived from the KOOS to expand the constructs measured from the WOMAC and to improve scale sensitivity and responsiveness in younger, more athletically active patients undergoing a THA for treatment of OA [1]. Lastly, authors of the HOOS constructed five additional items: two in the pain construct, two in the symptoms construct, and one in the sport and recreation construct.

In total, the HOOS assesses five dimensions of hip-related health with 40 items: Pain (10 items), Symptoms (5 items), Function limitations-daily living (17 items), Sport and Recreation Function (Sport = 4 items), and Hip Related QOL (QOL = 4 items) [1,4]. The scale uses five-option Likert-boxes with three different scale options (i.e., never to always, none to extreme, or never to constantly) across the five constructs [1,4]. All items are scored 0 to 4; each dimension is individually scored then transformed into a 0–100 scale [1,4], with 100 indicating no symptoms and 0 indicating extreme symptoms. The HOOS has been translated to 26 different languages and is recognized in the United States as an acceptable outcome for measuring functional assessment in patients 21 years of age and older who have been diagnosed with OA [5]. Although the HOOS was developed to assess outcomes in patients with OA, the HOOS is also used globally for reimbursement purposes to assess short-term and long-term changes induced by a variety of treatment options including, but not limited to, THA [4]. As such, it is pertinent to assess the measurement properties of the HOOS across diverse patient populations.

Early scale validation work has focused on validity (i.e., construct), reliability, and instrument responsiveness [1,6,7,8]. Construct validity of the HOOS was assessed by correlating the constructs to the SF-36, where moderate correlations (*r* = 0.49–0.66) were identified between constructs measuring physical health (i.e., function and pain); however, weaker correlations were identified for assessment of mental health (*r* = 0.26) [1]. Additionally, Cronbach’s alpha values for the constructs (e.g., pain, symptoms) of the HOOS ranged from 0.75 to 0.98 across multiple studies [6,7,8]. Values ranging from 0.70–0.89 have been generally recommended for each construct within an instrument [9,10,11]: (1) exceptionally high values (i.e., >0.90) may be indicative of item redundancy, parallel items, construct underrepresentation, inclusion of too many items, and reduced precision [11,12,13,14], and (2) low values (i.e., <0.70 in general; ≤0.80 for research tools) may indicate poor internal consistency within the instrument [9,10,12,13,14].

Test-retest reliability of the HOOS has also been reported, with values ranging from good to excellent (ICC = 0.75 to 0.97) [6,7,8]. Responsiveness (i.e., the validity of the HOOS over time) has also been established; the HOOS was significantly more responsive in the pain and symptoms constructs (SRM: 2.11, 1.83) compared to the pain and stiffness constructs of the WOMAC (SRM: 1.83, 1.28) [1]. Lastly, patients younger than 66 years of age reported a higher responsiveness in all five constructs of the HOOS compared to patients over the age of 66 [1].

Few researchers have examined the psychometric properties of the HOOS using exploratory factor analysis (EFA) or confirmatory factor analysis (CFA) and invariance procedures to verify the underlying factor structure and ensure measurement invariance as is recommended in scale development [8,9,10,11,12]. Minimal studies pertaining to CFA procedures have been published on individual constructs proposed in the original HOOS [15]. Previous authors performed CFAs on individual constructs and model fit recommendations were met for pain (i.e., CFI = 0.99, TLI = 0.98) and function (i.e., CFI = 0.97, TLI = 0.97); however, other recommended fit indices were not met (i.e., RMSEA = 0.14–0.19) for these constructs [15]. The full scale (i.e., all constructs) should also be assessed using factor analysis procedures, as recommended for best practices [9,10,12,13]. Researchers have reported that the full scale structure did not meet contemporary fit recommendations (CFI = 0.85; TLI = 0.84; IFI = 0.85; RMSEA = 0.10) in a sample of primarily self-reported healthy participants [16]. Further, correlations found between first-order latent constructs (e.g., pain, function) were high (ranging from *r* = 0.80–0.96); modification indices revealed several meaningful cross-loadings were present (e.g., putting on socks/stockings, taking off socks/stockings) and assessment of error-term correlations revealed that most of the items shared commonalities [16]. Overall findings of this study do not support the factorial validity of the original HOOS structure and suggest the presence of multicollinearity (i.e., overlapping items or items that are perceived to ask similar questions) and reduced measurement precision [10,11,12].

The reported poor psychometric properties of the HOOS were not surprising given that the scale is predominantly derived from the WOMAC, which has also been reported to have questionable psychometric properties. For example, poor fit indices values and error-term correlation findings on the HOOS [16] are similar to those found when examining the scale structure of the WOMAC [17,18]. Authors identified that the pain construct was not supported as a single factor with uncorrelated error-terms (CFI = 0.90; TLI = 0.80; RMSEA = 0.21), and the modification indices revealed significant error correlations between “at night while in bed” and “sitting or lying” and “walking on flat ground” and “going up or down stairs” [18]. Researchers examining the scale structure of the WOMAC performed a CFA on 11 of the 24 items (i.e., 3 pain items, 8 function items) and reported moderate overall fit in two samples (CFI = 0.95–0.97; RMSEA = 0.70–0.08) [17]. However, error-term correlations were specified between the items in this model, which included pain item 1 (i.e., walking on flat surface) and function item 6 (i.e., walking on flat surface), pain item 2 (i.e., up/down stairs) and function item 2 (i.e., ascending stairs), and function item 7 (i.e., getting in/out of a car) and function item 9 (i.e., putting on socks) [17]. The addition of error-term correlations between items limits the conclusions that can be drawn from the scale; as such, previous methods of scoring may not be sufficient as the items correlated cannot be scored separately [19]. Therefore, difficulties arise when trying to interpret the scoring for the instrument and is not recommended for best practices [12,19].

While the previous findings call into question the factorial validity of the HOOS and WOMAC, there were limitations noted in the previous studies worth considering. First, the CFAs were generally performed on individual constructs instead of examining the full factor structure as is recommended [11,12,20,21]. Second, one study included a CFA on the full model that utilized a moderate sample (n = 655) of mostly healthy respondents [16]. Further, invariance testing (e.g., multi-group, longitudinal) results have not been reported in the target population (i.e., THA patients); this testing is an important process that ensures the interpretations between groups (e.g., male vs. females) or across time (e.g., preoperative and postoperative visits) are valid and reliable [9,22].

Despite the current use of the HOOS (e.g., approved outcome measure for reimbursement purposes following THA), complete and robust psychometric analysis of the scale in a large dataset of patients for which the scale is designed has not been performed. As such, there is a need to conduct a CFA on the full scale to test the hypothesized factor structure of the 40-item HOOS to ensure that the items are appropriate indirect measures of the hypothesized latent constructs in a large, targeted sample of patients seeking care for the hip (e.g., THA) [21,22]. If the scale structure fails to meet recommended levels during the CFA procedures, alternate model generation should be conducted, following best practice recommendations [23], on the given items to determine if a parsimonious scale structure can be identified prior to further testing [12,21,22]. Further, there is need for invariance testing to ensure the scale is unbiased towards different groups of interest [12,21,22]. Lasty, it is important to understand how different groups respond to the outcomes over time postoperatively.

Therefore, the primary purpose of this study was to assess the psychometric properties of the HOOS in a large, diverse sample of patients who underwent a THA. Because model fit recommendations for the instrument were not met, alternate model generation procedures were performed to identify a more parsimonious model. The secondary purpose of this study was to conduct invariance testing between age groups and sex (i.e., multi-group), and longitudinal invariance (i.e., across time points), and to perform latent growth-curve (LGC) modeling on the parsimonious scale structure identified.

## 2. Materials and Methods

### 2.1. Data Source and Participants

Data were obtained for analysis from an international patient-reported outcomes (PRO) database (i.e., Surgical Outcome System [SOS]) global registry maintained by Arthrex (Arthrex, Inc., Naples, FL, USA). The SOS database was retrospectively queried to establish a large dataset for assessment of the psychometric properties of the 40-item HOOS. Patients who underwent a THA and completed the HOOS preoperatively and postoperatively were included in the sample. In addition, patient demographics that were queried included sex, age at treatment, race, and ethnicity. University Institutional Review Board (IRB) study was not required as this study was considered non-human subject research due to the de-identified nature of the data pull. However, prior IRB approval was granted as part of a larger research project at the host institution for collection and use of the SOS data.

### 2.2. Data Analysis

#### 2.2.1. Data Cleaning

Data were exported from the SOS database using Microsoft^®^ Excel for Mac (Version 16.46; Redmon, WA, USA). Once downloaded, the data were cleaned and analyzed using Statistical Package for Social Sciences (SPSS) version 28.0 (IBM Corp., Armonk, NY, USA). As the primary purpose was to assess the 40-item HOOS, individuals with missing demographic data were left as missing values and not excluded from analysis. Univariate outliers were assessed using z-scores, and removal was warranted if they exceeded the |3.3| cut-off value. In the presence of multivariate outliers, participants were assessed, flagged, and removed if Mahalanobis distance was exceeded [12,24]. Cut-off value was identified using a chi-square table with degrees of freedom (df = 40) and *p*-value < 0.01 [12,24].

#### 2.2.2. Confirmatory Factor Analysis

To assess the scale structure of the original 40-item HOOS, a CFA was performed with Analysis of Moment Structures (AMOS) software version 27 (IBM Corp., Armonk, NY, USA). The original HOOS was specified as a five-factor (i.e., Pain, Symptoms, ADLs, Sport, QOL), 40-item model to remain consistent with the original proposed model [1]. For generation of the parameter estimates, Full Information Maximum Likelihood estimation was used [12,20]. In addition, model fit statistics were assessed based on a priori values; the following goodness-of-fit indices were used: likelihood ratio statistic (CMIN), Comparative Fit Index (CFI) of ≥0.95, Goodness of Fit Index (GFI) of ≥0.95, Tucker-Lewis Index (TLI) of ≥0.95, Bollen’s Incremental Fit Index (IFI) of ≥0.95, and Root Mean Square Error of Approximation (RMSEA) of ≤0.05 [12,20,25]. In addition to assessing the overall goodness-of-fit, interpretability, size, and significance of the model’s parameter estimates (i.e., factor variances, covariances, indicator errors) were examined to identify any localized areas of strain [21].

#### 2.2.3. Alternate Model Generation

As model fit indices were not met, the dataset was split randomly into two samples for exploratory (i.e., sample n1) and confirmatory (i.e., sample n2) analyses. Exploratory factor analysis (EFA) was conducted in SPSS with sample n1 using Maximum Likelihood extraction and direct Oblimin Rotation to identify a parsimonious solution. To determine the number of factors to retain, the following criteria were used: (1) factors with an eigenvalue > 1.0, (2) scree plot inflexion point examination, and (3) factors that accounted for more than 5% of the variance [9,21,23,26]. To confirm the number of factors to obtain, parallel analysis was conducted [27]. Bartlett’s test for sphericity and Kaiser–Meyer Olkin (KMO) Measure of Sampling Adequacy were both assessed for violations. Cut-off values were set a priori at <0.01 for Bartlett’s test of sphericity and ≥0.80 for KMO [9]. Items were assessed individually and removed one at a time with the analysis being re-run with each item removal until a parsimonious scale structure was identified. Items with the greatest number of violations (e.g., those with loadings less than 0.40, multiple cross-loadings at 0.30 or greater, and poor theoretical fit) were removed first [9]. For analysis purposes, cross-loadings were defined as substantial (≥0.30) or extreme (≥0.45) [9,21]. Lastly, Cronbach’s alpha and McDonald’s Omega were assessed on each factor and set a priori between 0.70–0.89 [9,10,13].

#### 2.2.4. Confirmatory Factor Analysis of the Alternate Model

The parsimonious solution identified during the alternate model generation (i.e., EFA) process was then assessed using CFA procedures in AMOS with the sample n2. The same goodness-of-fit criteria that were utilized for the initial CFA were also used to assess model fit [12,21]. Modification indices, factor loadings, and correlations between constructs were also analyzed. Lastly, a correlational analysis was conducted on the total scores of the HOOS and the alternate HOOS to determine if the scale explained and acceptable amount of variance (*r* ≥ 0.90; R^2^ = 0.81) [28,29].

#### 2.2.5. Invariance Testing

Invariance testing was conducted using the full sample to determine if the association between the latent constructs and the associated items were stable and equal across groups (i.e., age and sex) and time points [12,20,21]. This process requires using a set of hierarchical steps with an increasing level of constraint [12,20,21]. Additionally, two types of invariance testing were conducted separately: (1) multi-group (i.e., age groups and sex) at time point 1 (i.e., preoperative visit), and (2) longitudinal across five time points (i.e., preoperatively (time point 1), 6 months postoperatively (time point 2), 1-year postoperatively (time point 3), 2-years postoperatively (time point 4), and 3-years postoperatively (time point 5). Individual CFAs were conducted by subgroup category and across each time point, ensuring the factors (e.g., pain, function, symptoms, etc.) were measuring what was intended among groups and across time [20,21]. Following the individual CFAs, the model then underwent configural, metric, and scalar invariance testing [12,20,21]. Configural invariance is first conducted, which places all subgroups or time points in the same model to ensure the same factors have similar items across the subgroups (e.g., males and females) or time points (e.g., time point 1 and time point 2). Second, the metric model determines if factor loadings were equal across groups and time points; invariance at this step would ensure that the meanings of the factors are similar between groups [20]. If metric model invariance requirements were met, equal variances (i.e., group differences and time differences) are assessed [20]. Lastly, the scalar invariance ensures that item intercepts are equal across groups and time points, which indicates the means were not determined or altered by external factors [20]. If scalar invariance requirements are met, equal mean models (i.e., score differences) are tested between groups and time points [20]. The chi-square difference test (χ^2^_DIFF_) and the CFI difference test (CFI_DIFF_) were used to compare model fit, with a *p*-value cut-off of 0.01 [20,25]. Due to the χ^2^_DIFF_ test being sensitive to sample size [25], the CFI_DIFF_ test held greater weight in decisions regarding invariance testing model fit. If a model exceeded the χ^2^_DIFF_ test but met the CFI_DIFF_ test, invariance testing proceeded.

#### 2.2.6. Latent Growth Curve Modeling

Full Information Maximum Likelihood was used when conducting the LGC modeling in AMOS [9]. We hypothesized the model to be linear (i.e., steady rate of change) [20], where perceived hip pain would decrease, and hip function would increase preoperatively to postoperatively following the intervention. Similar to the goodness-of-fit criteria for the CFA analyses, the same recommended cut-off values were used to assess the LGC model [9,20,21]. Specific to the model, two growth parameters were used, which included (1) the intercept parameter (i.e., patient’s score preoperatively (time point 1) and (2) slope parameter (i.e., patient’s rate of change across time points) [20]. Covariance was included between the intercept and slope factors to determine the rate of change between preoperative and postoperative scores [20]. The values assigned to the slope parameters were represented as the preoperative visit = 0, 6 months postoperatively = 0.5, 1-year postoperatively = 1, 2-year postoperatively = 2, and 3-year postoperatively = 3 [20,30,31]. If the growth trajectory was statistically significant (i.e., heterogeneous), groups including age and sex were then added to determine if the sample could be further explained [9,20]. If the model did not meet the cutoff values and a nonlinear growth model was identified, exploratory methods would be used to identify the shape of the model [20,30,31].

## 3. Results

A total of 6724 participants provided answers to all 40 HOOS items at the preoperative visit within the SOS database. Univariate (n = 148; 2.20%) outliers were identified at the preoperative visit in the 6724 cases and were subsequently removed. Deleted cases from the sample included both females (n = 68) and males (n = 80), with an average age of 67.36 ± 10.36 years (range = 42–87). To perform analyses, the data were randomly split into two equal samples (n1 = 3288, n2 = 3288) of cases. To determine a random selection of cases for splitting the sample (i.e., sample n1 and sample n2), a three-step process was used: (1) a unique number was created with a random number generator for each case, (2) cases were then sorted in ascending order by the unique identifier, and (3) the first 3288 cases were selected for sample n1, while the remaining 3288 cases were selected for sample n2. Sample n1 consisted of 48.10% males (n = 1582) and 51.70% females (n = 1701) with a mean age of 62.70 ± 11.17 years (range = 23–89 years). Sample n2 consisted of 48.10% males (n = 1582) and 51.70% females (n = 1701) with a mean age of 63.05 ± 10.69 years (range = 19–89 years). Lastly, to perform the longitudinal invariance testing, only those individuals who responded to the 40-item HOOS at all time points (i.e., preoperative and postoperative at 6-months, 1-year, 2-years, and 3-years) were included in the analysis. This sample consisted of 1144 patients (561 = males (49.00%), 580 = females (50.70%), 3 = missing (0.30%) with a mean age of 62.03 ± 9.83 y (range = 27–90 y).

### 3.1. Confirmatory Factor Analysis

**Figure 1:**
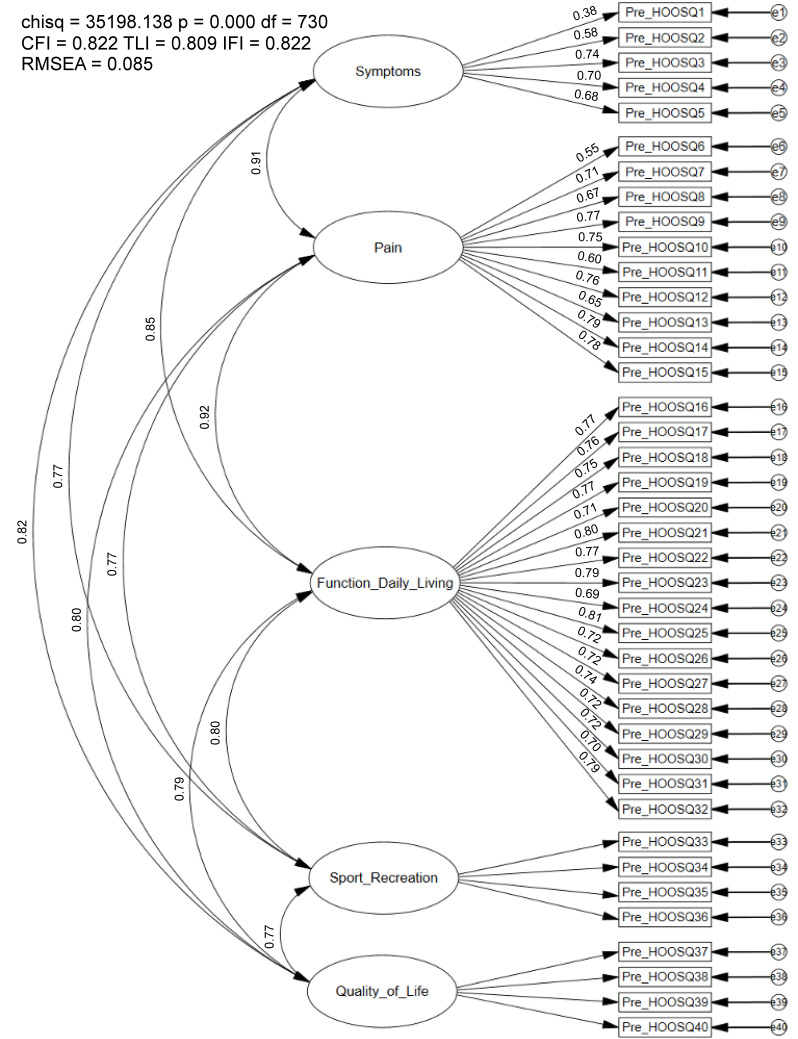
Confirmatory Factor Analysis of the 40-item HOOS. Chisq = Chi Square (χ^2^); df = degrees of freedom, *p* = alpha level; CFI = Comparative Fit Index; TLI = Tucker–Lewis Index; IFI = Bollen’s Incremental Fit Index; RMSEA = Root Mean Square Error of Approximation; SRMR = Standardized Root Mean Square Residual.

The CFA conducted on the final full sample (n = 6576) of the original five-factor, 40-item HOOS did not meet recommended model fit indices (CFI = 0.82, TLI = 0.81, IFI = 0.82, RMSEA = 0.09; Figure 1). Factor loadings were significant and ranged from 0.38–0.88. Correlations between first-order latent variables (e.g., symptoms, pain, ADLs) were high and ranged from *r* = 0.77–0.92. Modification indices revealed significant cross-loadings between multiple items (e.g., item 6 [how often is your hip painful]) and item 37 (i.e., how often are you aware of your hip problem; 536.30). Additionally, high error-term correlations were identified between several items (e.g., item 24 [putting on socks/stockings]) and item 26 (taking off sock/stockings; 2986.41). Therefore, the dataset was randomly split for further analyses due to poor model fit, multicollinearity between first-order latent variables, and item redundancy.

### 3.2. Alternate Model Generation

**Figure 2:**
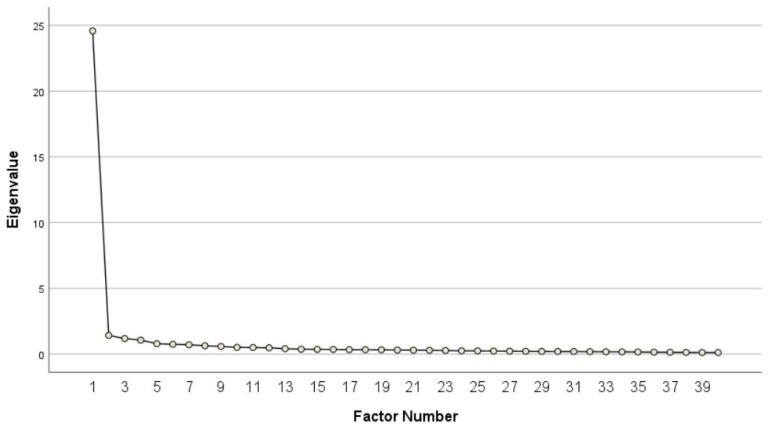
Scree plot of initial EFA Extraction of the 40-item HOOS in Sample n1.

Initial EFA of the original 40-item HOOS in sample n1 extracted four factors with eigenvalues > 1.0 (Figure 2) that accounted for 67.94% of the variance (Table 1). The parallel analysis, however, indicated only three factors should be retained; the fourth factor was slightly under the recommended cut-off when the raw data were compared to the percentile random data eigenvalue (Table 2). Following extraction, item loadings, cross-loadings, and analysis of item content were assessed individually throughout the process; 25 items had low loadings, substantial cross-loadings, or poor conceptual fit and were eliminated. An additional 7 items were removed (i.e., 31 items in total) due to high cross-loadings, low loadings, inflated high inter-item correlation values, or had overall lack of conceptual fit.

A three-factor, nine-item alternate HOOS (i.e., HOOS-9) was retained (Table 3). The solution accounted for 81.64% of the variance, contained items with loadings ≥ 0.48, and had Cronbach’s alpha and MacDonald’s Omega ranging from 0.86–0.89. Factor two and factor three accounted for more than 5% of the variance; however, the eigenvalues fell below the a priori cut-off of 1.00. Factor one contained the original HOOS items 17, 19, and 21 and assessed perceived function pertaining to daily living and were relabeled “ADLs”. Factor two contained the original HOOS items 33, 34, and 35, which measured difficulty pertaining to higher levels of activity, and retained the original label “Sport”. Lastly, factor three contained the original HOOS items 38, 39, and 40 that assess the patients’ perceived hip related QOL; therefore, the original label “QOL” was retained.

### 3.3. Confirmatory Factor Analysis of the Alternate Model

**Figure 3:**
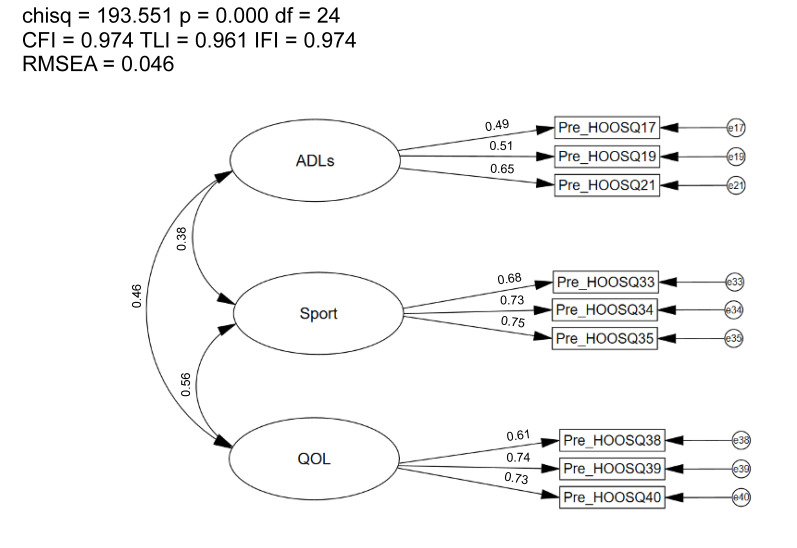
Confirmatory Factor Analysis in Sample n2 of the Modified 9-item HOOS. Chisq = Chi Square (χ^2^); df = degrees of freedom, *p* = alpha level; CFI = Comparative Fit Index; TLI = Tucker-Lewis Index; IFI = Bollen’s Incremental Fit Index; RMSEA = Root Mean Square Error of Approximation; SRMR = Standardized Root Mean Square Residual.

The CFA using sample n2 of the alternate model of the HOOS-9 had an improved fit (Figure 2), with the goodness-of-fit indices exceeding the recommended values (CFI = 0.97, TLI = 0.96, RMSEA = 0.05; Figure 3) [9]. Factor loadings were significant and ranged from 0.49–0.75. Correlations between first-order latent variables (i.e., ADLs, Sport, QOL) were improved and ranged from *r* = 0.38–0.56. Modification indices did not reveal any significant cross-loadings or error-term correlations. Lastly, participant scores from the original 40-item HOOS and the modified HOOS-9 were highly correlated between the function of daily living and ADLs constructs (*r* = 0.90, R^2^ = 0.80), sport constructs (*r* = 0.97, R^2^ = 0.94), and QOL constructs (*r* = 0.98, R^2^ = 0.96) [20,30].

### 3.4. Multi-Group Invariance Testing of the Alternate Model

#### 3.4.1. Sex Groups

The full sample was used to conduct invariance testing: of the 6566 individuals in the sample, 3164 (48.20%) were males and 3402 (51.80%) were females. Model fit indices were met (CFI = 0.98; χ^2^ = 699.88; RMSEA = 0.06; Table 4) for the configural model. Additionally, the metric model passed the CFI_DIFF_ (CFI = 0.98) and χ^2^_DIFF_ (χ^2^ = 31.67) tests. Because the metric model was considered invariant, the equal latent variable factors warranted examination. The CFI_DIFF_ (CFI = 0.98) and χ^2^_DIFF_ (χ^2^ = 42.67) tests passed for the equal factor variances model. The scalar model passed the CFI_DIFF_ test (CFI = 0.98) and the χ^2^_DIFF_ test (χ^2^ = 116.03). Examination of the latent means model was warranted because the scalar model passed the CFI_DIFF_ and the χ^2^_DIFF_ tests, and the equal latent means model passed the CFI_DIFF_ (CFI = 0.98) and χ^2^_DIFF_ (χ^2^ = 201.43) tests.

#### 3.4.2. Age Groups

Patients who reported their age were included in the analysis and grouped by the following age ranges: <45 y (n = 374; 5.69%), 45–54 y (n = 1043; 15.86%), 55–64 y (n = 2200; 33.45%), 65–74 y (n = 1983; 30.16%), and ≥75 y (n = 976; 14.84%). The configural model met all model fit indices (CFI = 0.98; χ^2^ = 801.94; RMSEA = 0.03; Table 5). In addition, the metric model passed the CFI_DIFF_ (CFI = 0.98) and χ^2^_DIFF_ (χ^2^ = 41.39) tests. Because the metric model was considered invariant, further examination was warranted with testing of the equal latent variable factors. The equal factor variance model passed the CFI_DIFF_ (CFI = 0.98) and χ^2^_DIFF_ (χ^2^ = 99.43) tests. The scalar model also passed the CFI_DIFF_ (CFI = 0.97) and χ^2^_DIFF_ (χ^2^ = 248.67) tests. Lastly, examination of the latent mean model was warranted as the scalar model was invariant. The equal latent means model passed the CFI_DIFF_ (CFI = 0.97) and the χ^2^_DIFF_ (χ^2^ = 301.23) tests.

### 3.5. Longitudinal Invariance Testing of the Alternate Model

Of the total 6576 patients included in the full sample, 1144 (17.40%) patients responded to the HOOS at all five time points (i.e., preoperatively, 6-months postoperatively, 1-year postoperatively, 2-years postoperatively, 3-years postoperatively). The configural model met all model fit indices (CFI = 0.98; χ^2^ = 1511.52; RMSEA = 0.03; Table 6). The metric model passed the CFI_DIFF_ (CFI = 0.98) and χ^2^_DIFF_ (χ^2^ = 57.53) tests. Testing of the equal latent variable factors was performed because the metric model was invariant. The equal factor variance model passed the CFI_DIFF_ (CFI = 0.97) and the χ^2^_DIFF_ (χ^2^ = 262.11) tests. Also, the scalar model passed the CFI_DIFF_ (CFI = 0.97) and χ^2^_DIFF_ (χ^2^ = 299.08) tests. As the scalar model passed the CFI_DIFF_ and the χ^2^_DIFF_ test, evaluation of the equal latent means model was warranted. The equal latent means model did not pass the CFI_DIFF_ (CFI = 0.91) or χ^2^_DIFF_ (χ^2^ = 2504.84) tests. When the means were not constrained to be equal, the scores significantly improved at each time point, indicating less hip pain and disability, with time point 5 having an overall lower score compared to baseline.

### 3.6. Latent Growth-Curve Model of the Alternate Model

Patients who answered the HOOS at all five time points (n = 1144) were included in the analyses. Scores of the alternate HOOS-9 were calculated by averaging the 9 items, dividing by 4, multiplying by 100, then subtracting the total from 100. These calculation guidelines are similar to the guidelines published by previous authors [32]; however, a total score was calculated for the alternate HOOS-9 rather than individual construct scores (i.e., ADLs, Sport, QOL). In addition, a bifactor model was assessed to determine if the scale could be summed as a total score rather than the individual construct scores [20]. Goodness-of-fit indices met the recommended criterion (CFI = 0.99, TLI = 0.99, IFI = 0.99, RMSEA = 0.03) [20]. Therefore, we proceeded with the total summed score for assessment of LGC modeling.

**Figure 4:**
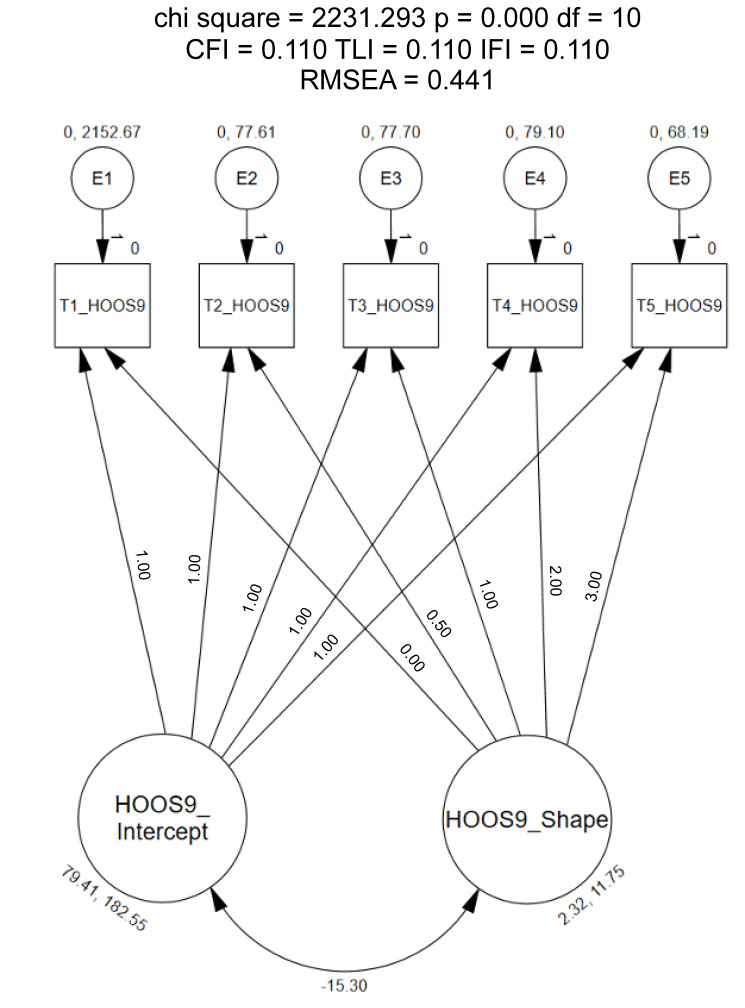
Linear Latent Growth Model of the 9-item HOOS. Chisq = Chi Square (χ^2^); df = degrees of freedom, *p* = alpha level; CFI = Comparative Fit Index; TLI = Tucker–Lewis Index; IFI = Bollen’s Incremental Fit Index; RMSEA = Root Mean Square Error of Approximation.

When defined as linear, the LGC model did not meet the recommended fit indices (CFI = 0.11, TLI = 0.11, IFI = 0.11, RMSEA = 0.44; Figure 4) [20]. These findings demonstrate that patients’ response scores increase over each time point (i.e., hip disability improving over time); however, the change in scores did not increasing at a consistent rate over each visit. Due to these findings, exploratory methods were used to identify the shape of the growth trajectory. First, the slope parameters were constrained to 0 (time point 1) and 0.5 (time point 2). The remaining time points were freely estimated [20,33].

**Figure 5:**
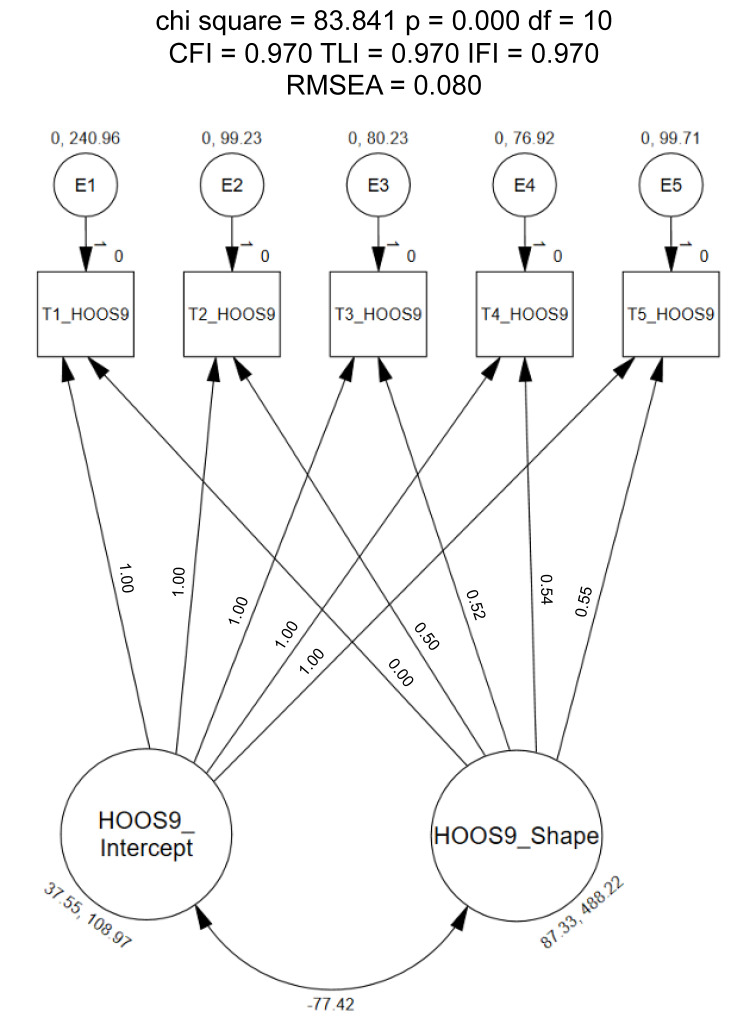
Exploratory Latent Growth Model of the 9-item HOOS. Chisq = Chi Square (χ^2^); df = degrees of freedom, *p* = alpha level; CFI = Comparative Fit Index; TLI = Tucker–Lewis Index; IFI = Bollen’s Incremental Fit Index; RMSEA = Root Mean Square Error of Approximation.

Upon assessment of these findings, the final slope parameters were defined as follows: (1) preoperatively = 0, (2) 6-months postoperatively = 0.50, (3) 1-year postoperatively = 0.52, (4) 2-years postoperatively = 0.54, and (5) 3-years postoperatively = 0.55. When the parameters were constrained to the prior definitions, the model met most recommended model fit indices (CFI = 0.97, TLI = 0.97, IFI = 0.97; Figure 5); however, the RMSEA was slightly exceeded (0.08). Parameters for both the intercepts and the shape were statistically significant (*p* < 0.001) upon assessment of the means estimates. Additional findings revealed the average score for the alternate HOOS-9 to be 37.55 points at time point 1 and that scores increased over the 3-year period to 87.33 points. A negative estimate was identified when assessing the covariance between the intercept and shape (i.e., −77.42). Upon assessment of the model estimates, the variances were not statistically significant for intercept (*p* = 0.13) or shape (*p* = 0.06).

Model fit indices were also assessed between sex and age groups to determine the differences these groups have on the mean scores and growth trajectories over time. When assessing the LGC model pertaining to sex, the model met all recommended fit indices (CFI = 0.97, TLI = 0.97, IFI = 0.97, RMSEA = 0.06). Estimates of the means for both the intercept and the shape were statistically significant (*p* < 0.001). The average mean scores for the alternate HOOS-9 at the preoperative visit was higher in patients within the male group (40.63) compared to patients in the female group (34.56). Patients in both groups improved their scores over the 3 years, with patients in the female group having an overall higher change in scores (i.e., females = 92.86, males = 91.75). Upon assessment of the covariance between the intercept and shape, a negative estimate was identified for patients in the male group (i.e., −67.61) and female group (−52.41; Table 7). Lastly, upon assessment of the variances, estimates were not statistically significant for the intercept (*p* = 0.28) or shape (*p* = 0.20).

The LCG model assessing age groups met all recommended fit indices (CFI = 0.97, TLI = 0.98, IFI = 0.97, RMSEA = 0.04). When assessing the means for the intercept and the shape, the estimates were statistically significant (*p* < 0.001). On average, patients in the age group 45–54 had an overall lower mean score at the preoperative visit (33.42) compared to other age groups (Table 7). All patients, regardless of age group, reported increased scores over the 3 years; however, patients in the 45–54 group had an overall higher change in scores (91.90). Upon assessment of the covariance between the intercept and shape, those <45 years of age had a large negative estimate (−461.30) compared to the other age groups (Table 7). Estimates pertaining to the variances were not significantly different for the intercept (*p* = 0.34) or shape (*p* = 0.19).

## 4. Discussion

With the occurrence of THAs expected to significantly increase by 2050 [34], it is imperative that clinicians have access to PROs that can be widely used across different sexes (i.e., males and females), age groups (i.e., 18–94), and repeated visits. Having a PRO to assess the patient’s perspective of hip health throughout their recovery is beneficial to clinicians to ensure positive outcomes following arthroplasty. More recently, significant attention has been focused on PROs associated with outcomes following THA [35,36]. Therefore, the need to establish a psychometrically sound tool that adequately measures the multifaceted nature of hip pain and function is valuable. Previous psychometric analysis on the HOOS has yet to yield a scale structure that meets recommended model fit indices [16], and assessment of how age and sex influence patient responses to the individual items and mean scores has not been conducted [1,16,37] As such, the primary purpose of this study was to assess the psychometric properties of the original 40-item HOOS in patients undergoing THA. The CFA of the 40-item HOOS did not meet recommended model fit indices. Therefore, an EFA was conducted to establish a more parsimonious scale structure. The alternate three-factor, nine-item (HOOS-9) was then subjected to multi-group invariance testing (i.e., sex and age groups), longitudinal invariance testing across five time points (i.e., preoperatively, 6-months postoperatively, 1-year postoperatively, 2-years postoperatively, 3-years postoperatively), and LGC modeling across five time points. The alternate HOOS-9 met recommended measurement criteria and can be recommended for use in research and clinical practice.

### 4.1. Confirmatory Factor Analysis

The original five-factor, forty-item scale structure of the HOOS was not supported in our study, as demonstrated by the poor model fit indices and the high latent variable correlations. However, our findings are consistent with previous research where a well-supported scale structure in mostly healthy adults was not found [16]. High to very high correlations (*r* = 0.77–0.91) between the first-order latent variables were found, indicating multicollinearity between factors. Modification indices also demonstrated there were items with meaningful cross-loadings, indicating overlapping items were present (e.g., item six (how often is your hip painful) and item 37 (how often are you aware of your hip problem) and high error-term correlations between several items (e.g., item 24 [putting on socks/stockings] and item 26 [taking off socks/stockings]). These findings further suggest the presence of multicollinearity. Poor model fit and the presence of multicollinearity provides evidence that the current 40-item scale should not be used [12,21,38]. Thus, to determine if a psychometrically sound version could be identified using the original items, alternate model generation was warranted [12,21,38].

### 4.2. Psychometric Analysis of the Alternate HOOS-9

An EFA was conducted using a calibration sample (i.e., sample n1), which yielded an alternate three-factor, nine-item solution (i.e., HOOS-9). The nine items represented three of the original five constructs of the HOOS: three items from “Function, daily living” (i.e., original HOOS items 17, 19, 21), three items from “Function, sports and recreational activities” (i.e., original HOOS items 33, 34, 35), and three items from “Quality of Life” (i.e., original HOOS items 38, 39, 40). The alternate HOOS-9 underwent covariance modeling procedures using the validation sample (i.e., sample n2). As the alternate HOOS-9 only retained 22.50% of the questions from the original scale, participant responses were highly correlated (*r* = 0.93) with the original 40-item HOOS and accounted for a substantial amount of the variance (R^2^ = 0.87).

The three-factor structure identified in our sample is different than other HOOS short-forms, including the HOOS-JR (i.e., original HOOS items 18, 15, 18, 20, 27, 29), HOOS-PS (i.e., original HOOS items 29, 16, 28, 34, 35), and more specifically the three-factor, twelve-item HOOS (HOOS-12) [15,39]. The HOOS-12 is short-form version of the original 40-item HOOS that includes three-factors (i.e., Pain, Function daily living, and QOL) consisting of twelve items (i.e., original HOOS items 6, 9, 10,12, 18, 19, 22, 36–40); however, our alternate HOOS-9 model contains four items present in the HOOS-12 (i.e., original HOOS items 19, 38, 39, 40) in the ADLs and QOL construct. When developing the HOOS-12, authors used computerized adaptive test (CAT) simulations to identify items to best match patients’ level of pain and function [39]. Limitations exist with the use of CAT such as high cost and the adaptability of the questionnaire to the individual persons responses [40]. Therefore, patients may not be answering the same questions based on their responses to the bank of items. This methodology poses further limitations on the ability of clinicians attempting to draw conclusions of the PROs; as such, CAT may not always be appropriate in the clinical setting [40]. Additional assessment of structural validity on the HOOS-12 was conducted by performing CFAs on the individual constructs (i.e., pain, function, QOL) and not on the full scale [15]. Best practice recommendations when performing CFA is to assess the entire scale and, if the model meets recommended fit indices, perform invariance testing (e.g., multi-group, longitudinal) to ensure the instrument can be used across several groups and time points [12,20,21].

In addition to these findings, previous research assessing structural validity using CFA on the full HOOS-12 did not support its use in a sample of mostly healthy adults [16]. Several concerns related to the scale were noted: high correlations between the constructs (i.e., indicating potential multicollinearity), high Cronbach’s alpha values (i.e., indicating potential item redundancy), and cross-loadings of items (i.e., items shared commonalities) [16,21]. Therefore, further testing of the HOOS-12 was not warranted in the population studied [16]. In our identified model, correlations between constructs ranged from 0.38–0.56, and modification indices did not reveal cross-loadings between items. Therefore, our findings present a newly refined short-form version of original HOOS items that measures unique constructs.

### 4.3. Multi-Group and Longitudinal Invariance Testing of the Alternate HOOS-9

We assessed group differences using CFA methods between groups of interest (i.e., age groups and sex) and across several time points for the alternate HOOS-9. Invariance testing confirms the structural validity of the scale, ensuring the association between constructs (i.e., ADLs, Sport, and QOL) are being measured and their items are being interpreted similarly across groups (i.e., males, females) and time (i.e., multiple visits) [12,20,21]. Thus, an invariant instrument allows clinicians to compare scores across groups or visits and provides support that score differences in hip health are true group differences as opposed to measurement errors [12,21]. Minimal studies exist assessing multi-group and longitudinal invariance using any versions of the HOOS; previous work has focused on invariance testing pertaining to multiple short-forms (i.e., the HOOS-JR and HOOS-PS) [16]. In a previous study, however, differences between hip pathology and physical activity groups in the HOOS-JR and HOOS-PS were assessed [16]. In a more recent study, multi-group (i.e., age groups and sex) and longitudinal invariance (i.e., multiple visits) in a similar sample of patients who underwent a THA was also conducted. To our knowledge, this was only the second study to assess multi-group and longitudinal invariance in a short-form version (i.e., HOOS-9) using items from the original 40-item HOOS.

We found the alternate HOOS-9 was invariant at the preoperative visit (i.e., preoperative THA) between age groups (i.e., <45, 45–54, 55–64, 65–74, ≥75) and sex (i.e., males, females). These results indicate that the new alternate scale can be used to assess differences in hip-related dysfunction in patients undergoing THA. In addition to our invariant findings, significant latent variances and latent mean differences were not found between age groups or sex, suggesting that minimal differences in hip disability were perceived between groups. These findings are different from those found in previous research, where latent mean differences in sex groups were found with females reporting higher mean scores on the HOOS-JR compared to males. In addition, other researchers identified sex and age differences, with females and those in older age groups reporting higher scores on the 40-item HOOS and HOOS-12 for all domains [41,42]. However, Sunden et al. only identified significant differences between males and females in the oldest age group (i.e., 75–84); no significant differences in mean scores were found between males and females in different age groups (i.e., 18–35, 3–54, 55–74) [41]. Larsen et al. found significantly worse HOOS and HOOS-12 scores with increasing age. Within our population, the majority of our sample was younger than 75 years of age, which could partially explain these findings [42]. Of important note, these findings are associated with different versions of the HOOS scale, which include different items. Having different items compared to the other versions indicates that the scales are not necessarily measuring hip disability in the exact same way. Therefore, our findings are unique in that the scale structure of the alternate HOOS-9 demonstrates no significant differences between sex and age groups.

This study also provides evidence of scale validity of the alternate HOOS-9 for assessing postoperative effects across time. Longitudinal invariance was established across multiple visits (i.e., preoperatively and 6-months, 1-year, 2-years, and 3-years postoperatively), indicating that the scale can be used to assess differences in hip disability across time. Thus, the results supported the assessment of mean scores across time to determine if scores changed post-THA. We identified significant latent mean differences across time points, indicating that patients reported a meaningful improvement in scores preoperatively to 3-years postoperatively. In addition, the highest scores (i.e., more hip disability) were reported preoperatively and the lowest scores (i.e., less hip disability) were identified at 3-years postoperatively. These findings provide support for scale validity as patients who receive surgery would be expected to report improvement over time following the intervention (i.e., THA) as natural healing occurs across visits. These findings are congruent with previous research reporting significant improvement in scores on the HOOS and HOOS-12 in patients who underwent THA from preoperatively to 2-years postoperatively [43,44].

### 4.4. Alternate HOOS-9 Latent Growth-Curve Modeling

To our knowledge, this was the first study to perform LGC modeling in patients who answered questions of the 40-item HOOS over a 3-year period postoperatively. Use of LGC modeling is a robust technique that allows researchers to assess between-person differences and within-person change, which is unique compared to traditional longitudinal assessments (e.g., repeated-measures analyses or multivariate analyses) [20,45]. In addition, LGC modeling is highly flexible when attempting to assess differences in unequally spaced time points (e.g., months, years) and for more complex nonlinear data [20,45]. Few studies identified assessed outcomes related to hip disability (i.e., HOOS Physical Function [HOOS-PS], Oxford Hip Score [OHS]) over a 12-month period postoperatively in patients who underwent THA [46,47]. In addition, other researchers assessed LGC of the OHS in patients over 6-weeks postoperatively [48]. These three studies all identified a nonlinear improvement, with most improvement occurring within the first 6-weeks to 3-months postoperatively [46,47,48]. These findings are similar to ours; the lack of fit within the linear model, along with the re-defined nonlinear model, demonstrates the majority of the growth and improvement in scores occurred within the first 6-months postoperatively.

Researchers defined groups by healing trajectories (e.g., fast starters, early recovery) [47,48], PROs (i.e., OHS, HOOS-PS), or how the patients scored (i.e., high-high, intermediate, low-high) [46]. These defined groups differ from our study, which examines the differences age groups and sex have on responses to the alternate HOOS-9 over time. Our results indicate that patients in the male group have an overall higher score at baseline (40.63) compared to those in the female group (34.56). In addition, patients in both male and female groups who scored lower at baseline had an overall faster growth over time, although those in the female group had a slower rate of growth over time in comparison to males (−52.41 vs. −67.61), respectively. These findings are similar to Hesseling et al., who reported females were considered slow starters, meaning they had slower improvement in hip function and QOL within the first 3-months postoperatively but had a significant overall improvement at 1-year postoperatively [47]. In addition, we found females had a higher mean score at 3-years postoperatively (92.86) in comparison to males (81.75). However, even though differences were identified between patients in the male and female groups, the variances of the model for the intercept and shape were not statistically significant. This finding indicates that there were no significant differences between the two groups (i.e., interindividual differences).

To our knowledge this was also the first study to assess different age groups across time points. When assessing these differences, patients in the age group 45–54 scored the lowest overall at baseline (33.42) when compared to the other groups, and patients in the age group ≥75 had the highest overall mean score at baseline (41.12). In addition, patients in the age group >45 who had lower self-perceived hip function and QOL reported greater improvements in their scores (−461.30) compared with those in the aged 45–54 (−103.42) and 65–74 (−342.90); however, they had a slower rate of increase in scores over time. Patients in the ≥75 group had a steeper growth and improvement in HOOS-9 scores (451.13) over time, though had an overall lower mean score at time 5 (82.67) in comparison to those in the age group 55–64 (133.96). These findings indicate that patients in the age group ≥ 75 improve their hip disability and QOL faster but have an overall lower score on the alternate HOOS-9 compared to the other age groups. Variances between the intercept and slope, however, were not statistically significant (*p* > 0.05), which indicate interindividual differences are homogenous rather than heterogeneous.

### 4.5. Limitations and Future Research

Though this study contained a large sample of patients undergoing a THA, limitations are present that should be addressed. Though a cross-validation sample was used to assess the alternate HOOS-9, participants responded to the original 40-item HOOS. As such, the responses to items on the alternate HOOS-9 could be influenced by the other 31 items [38]. Therefore, future research should assess the scale structure on patients who only answer the nine items [38]. We assessed concurrent validity (i.e., correlation between two scales) between the original constructs of the 40-item HOOS and the newly proposed scale. Future researchers may want to consider conducting further analyses that correlate the HOOS-9 responses with other scales designed to measure similar dimensions (e.g., QOL). As this is the first study to report the HOOS-9, limitations may exist when attempting to assess differences in clinical practice and research. Therefore, future research should be conducted to determine the responsiveness, minimal clinically important difference, and reliability of the instrument.

Additionally, even though the HOOS-9 was invariant between groups of interest (i.e., sex and age groups), our dataset was limited and did not include other pertinent information, such as demographic data (e.g., race, ethnicity, medical history), diagnosis (e.g., osteoarthritis, hip dysplasia), surgical procedure (i.e., primary, revision), or operative data (e.g., surgical approach, laterality, implant type). Thus, caution is warranted when examining alternate HOOS-9 differences in groups that have not yet been analyzed. Future research should focus on invariance testing modeling across several different groups (e.g., diagnosis, surgical approach, surgical procedure) to ensure the scale has the necessary properties to support between groups analysis in these populations. Also, it may be pertinent for the development of a database that allows for collection of outcomes and patient information longitudinally. This would allow researchers to track outcomes associated with the surgical procedure and potentially identify populations in need of further medical care (e.g., infections, revisions) to determine if further scale refinement or creation of a new scale is needed in such populations. In addition, further analyses using LGC modeling with these different groups could help clinicians and researchers understand healing differences over time.

Another limitation of this study was the decision to score the alternate HOOS-9 as a total score versus scoring each construct individually for purposes of LGC modeling. Scoring PROs as a total score is common practice for documentation purposes to be able to easily assess changes over time. Our model fit statistics had low to moderate correlations between the first-order latent variables, thus providing justification that the items are measuring unique constructs. However, we performed a bifactor model to determine if a composite score could be used even though the constructs were unique. Our findings reveal acceptable goodness-of-fit indices, indicating that clinicians may be able to score the alternate HOOS-9 as a total summed score. Therefore, future research should be conducted to assess the reliability and validity (e.g., responsiveness) of the alternate HOOS-9 using the total summed scores. Although we had an overall large sample for this study, the sample size was much smaller (n = 1140) when assessing invariance and differences over time (i.e., longitudinal invariance and LCG modeling) due to the low percentage (17.40%) of patients who answered the items over all time points. Therefore, future research should be conducted in a larger sample to ensure similar findings exist.

## 5. Conclusions

The original scale structure of the 40-item HOOS was not supported in our study. We subsequently identified an alternate three-factor, 9-item HOOS (i.e., alternate HOOS-9) that met contemporary model fit recommendations, along with multi-group and longitudinal invariance testing. Our findings support the use of the alternate HOOS-9 as a more viable option to assess hip pain and disability. However, caution is warranted until assessment of the measurement properties is further conducted.

## Figures and Tables

**Table 1:** Initial EFA Extraction of the 40-item HOOS in Sample n1.

Item	Factor 1	Factor 2	Factor 3	Factor 4
Pre_HOOSQ21	**0.921**			
Pre_HOOSQ14	**0.904**			
Pre_HOOSQ9	**0.872**			
Pre_HOOSQ19	**0.740**			
Pre_HOOSQ15	**0.670**			
Pre_HOOSQ23	**0.667**			
Pre_HOOSQ12	**0.645**			
Pre_HOOSQ16	**0.594**			
Pre_HOOSQ10	**0.570**			
Pre_HOOSQ17	**0.562**			
Pre_HOOSQ32	**0.561**			
Pre_HOOSQ36	**0.522**			0.351
Pre_HOOSQ3	**0.436**			0.356
Pre_HOOSQ26		**0.778**		
Pre_HOOSQ24		**0.772**		
Pre_HOOSQ30		**0.524**		
Pre_HOOSQ20		**0.500**		
Pre_HOOSQ22		**0.477**		
Pre_HOOSQ25	0.313	**0.466**		
Pre_HOOSQ28	0.326	**0.420**		
Pre_HOOSQ18		**0.377**		
Pre_HOOSQ13			**−0.837**	
Pre_HOOSQ11			**−0.808**	
Pre_HOOSQ29			**−0.518**	
Pre_HOOSQ27		0.336	**−0.513**	
Pre_HOOSQ5			**−0.472**	
Pre_HOOSQ7	0.36		**−0.399**	
Pre_HOOSQ6			**−0.364**	
Pre_HOOSQ4			**−0.361**	
Pre_HOOSQ2			**−0.355**	0.35
Pre_HOOSQ8			**−0.314**	
Pre_HOOSQ34				**0.659**
Pre_HOOSQ35				**0.597**
Pre_HOOSQ33		0.31		**0.547**
Pre_HOOSQ38				**0.518**
Pre_HOOSQ39				**0.451**
Pre_HOOSQ40				**0.449**
Pre_HOOSQ37				**0.403**
Pre_HOOSQ31	0.304			**0.401**
Eigenvalue	24.57	1.42	1.18	1.06
% Variance	61.43	3.56	2.96	2.66

**Table 2:** Parallel Analysis of Raw Data Eigenvalues, Means, and Percentile Random Data Eigenvalues.

Number of Items	Raw Data	Means	Random Data
1	24.57 *	1.21	1.23 *
2	1.42 *	1.19	1.21 *
3	1.18 *	1.17	1.18 *
4	1.06	1.16	1.17
5	0.79	1.15	1.16
6	0.75	1.13	1.15
7	0.71	1.12	1.13
8	0.63	1.11	1.12
9	0.58	1.10	1.11
10	0.51	1.09	1.10

* *p* < 0.05; Note: Table only presents data for the first 10 of 44 items.

**Table 3:** Alternate HOOS-9 Exploratory Factor Analysis.

Item	Factor 1	Factor 2	Factor 3
Pre_HOOSQ17	0.747		
Pre_HOOSQ19	0.944		
Pre_HOOSQ21	0.889		
Pre_HOOSQ33		0.762	
Pre_HOOSQ34		0.988	
Pre_HOOSQ35		0.747	
Pre_HOOSQ38			0.970
Pre_HOOSQ39			0.843
Pre_HOOSQ40			0.575
Eigenvalue	5.84	0.84	0.67
% Variance	64.89	9.29	7.46
Cronbach Alpha	0.89	0.86	0.88
MacDonald’s Omega	0.89	0.86	0.88

Extraction Method: Maximum Likelihood; Rotation Method: Oblimin with Kaiser Normalization.

**Table 4:** Goodness-of-Fit Indices for Multi-Group Invariance Across Sex.

Modified 9-Item HOOS	χ^2^	df	χ^2^_DIFF_ (df_diff_)	CFI	CFI_diff_	TLI	RMSEA
Males (n = 3164)	332.98	24	----	0.979	----	0.969	0.064
Females (n = 3402)	366.90	24	----	0.977	----	0.966	0.065
Configural (equal form)	699.88	48	----	0.978	----	0.967	0.045
Metric (equal loadings)	731.55	54	31.67 (6)	0.977	0.001	0.970	0.044
Equal factor variances *	742.55	57	42.67 (9)	0.977	0.001	0.971	0.043
Scalar (equal indicator intercepts)	815.91	60	116.03 (12)	0.975	0.003	0.970	0.044
Equal latent means *	853.31	63	201.43 (15)	0.975	0.006	0.972	0.044

* = Substantive questions; ---- = difference test not performed.

**Table 5:** Goodness-of-Fit Indices for Multi-Group Invariance Across Age.

Modified 9-Item HOOS	χ^2^	df	χ^2^_DIFF_ (df_diff_)	CFI	CFI_diff_	TLI	RMSEA
<45 (n = 374)	77.48	24	----	0.966	----	0.950	0.080
45–54 (n = 1043)	167.60	24	----	0.968	----	0.952	0.076
55–64 (n = 2200)	232.38	24	----	0.980	----	0.971	0.063
65–74 (n = 1983)	197.52	24	----	0.980	----	0.971	0.060
≥75 (n = 976)	126.86	24	----	0.977	----	0.965	0.066
Configural (equal form)	801.94	120	----	0.977	----	0.966	0.029
Metric (equal loadings)	843.33	144	41.39 (24)	0.977	0.000	0.971	0.027
Equal factor variances *	901.37	156	99.43 (36)	0.975	0.002	0.971	0.027
Scalar (equal indicator intercepts)	1050.61	177	248.67 (57)	0.971	0.006	0.974	0.026
Equal latent means *	1103.17	180	301.23 (60)	0.969	0.008	0.969	0.028

* = Substantive questions; ---- = difference test not performed.

**Table 6:** Goodness-of-Fit Indices for Longitudinal Invariance Across Time Points.

Modified 9-Item HOOS	χ^2^	df	χ^2^_DIFF_ (df_diff_)	CFI	CFI_diff_	TLI	RMSEA
Preoperative	119.60	24	----	0.984	----	0.976	0.059
6-month postoperative	128.83	24	----	0.981	----	0.971	0.062
1-year postoperative	139.98	24	----	0.981	----	0.971	0.065
2-year postoperative	138.27	24	----	0.982	----	0.973	0.065
3-year postoperative	124.75	24	----	0.985	----	0.977	0.061
Configural (equal form)	1511.52	750	----	0.979	----	0.973	0.030
Metric (equal loadings)	1560.86	774	57.53 (24)	0.979	0.000	0.973	0.030
Equal factor variances *	1773.63	786	262.11(36)	0.973	0.006	0.966	0.033
Scalar (equal indicator intercepts)	1810.65	798	299.08 (48)	0.973	0.006	0.966	0.033
Equal latent means *	4016.36	810	**2504.84 (60)**	0.913	**0.066**	0.894	0.059

* = Substantive questions; **Bolded** = did not meet cuff off criteria; ---- = difference test not performed.

**Table 7:** Goodness-of-Fit Indices for Multi-Group Latent Growth-Curve Model.

Modified 9-Item HOOS	χ^2^	df	CFI	TLI	RMSEA	Intercept Mean	Covariance
Males (n = 561)	40.89	10	0.976	0.976	0.074	40.63	−67.61
Females (n = 580)	53.35	10	0.963	0.963	0.087	34.56	−52.41
>45 (n = 48)	12.17	10	0.989	0.989	0.068	34.44	−461.30
45–54 (n = 205)	15.16	10	0.990	0.990	0.050	33.42	−103.42
55–64 (n = 406)	33.03	10	0.974	0.974	0.075	38.38	133.96
65–74 (n = 375)	40.72	10	0.959	0.959	0.091	38.46	−342.90
≥75 (n = 110)	17.89	10	0.955	0.955	0.085	41.12	451.13

## Data Availability

The datasets analyzed during the study are not publicly available per study protocol. De-identified data may be available from the corresponding author with permission from the Cedar-Sinai Office of Research Compliance and Quality Improvement, the Kerlan-Jobe Institute, and the University of Idaho upon reasonable request.
